# CircASH2L facilitates tumor-like biologic behaviours and inflammation of fibroblast-like synoviocytes via miR-129-5p/HIPK2 axis in rheumatoid arthritis

**DOI:** 10.1186/s13018-021-02432-3

**Published:** 2021-05-08

**Authors:** Xia Li, Meiting Qu, Jie Zhang, Kuanyin Chen, Xianghui Ma

**Affiliations:** 1 Department of Nephrology, Western Theater General Hospital, Chengdu, Sichuan China; 2 Department of Pathology, Weifang Traditional Chinese Hospital, Weifang, Shandong China; 3 Department of Ophthalmology, Weifang Eye Hospital, Weifang, Shandong China; 4 Department of Critical Care Medicine, Traditional Chinese Medicine University of Guangzhou First Affiliated Hospital, Guangzhou, Guangdong China; 5 Department of Rheumatism, Dongying City People’s Hospital, No. 317, Nanyi Road, Dongying City, Shandong Province China

**Keywords:** circASH2L, RA, Fibroblast-like synoviocytes, miR-129-5p, HIPK2

## Abstract

**Background:**

Previous study showed that circular RNA Absent-Small-Homeotic-2--Like protein (circASH2L) was higher in rheumatoid arthritis (RA) patients. However, the roles and mechanisms of circASH2L in RA progression remain unclear.

**Methods:**

Levels analysis was conducted using western blot and qRT-PCR. The proliferation, apoptosis, cell cycle progression, migration, invasiveness, and inflammation of RA fibroblast-like synoviocytes (RA-FLSs) were determined via MTT, flow cytometry, western blot, transwell, and ELISA assays.

**Results:**

CircASH2L knockdown in RA-FLSs suppressed cell proliferative, migratory, and invasive capacities, triggered cell cycle arrest, promoted apoptosis, and inhibited inflammation. Mechanistically, circASH2L targeted miR-129-5p, and repression of miR-129-5p abolished the functions of circASH2L silencing on the growth, motility, and inflammation of RA-FLSs. Besides, miR-129-5p was found to directly target HIPK2, and suppressed the tumor-like biologic behaviors and inflammation of RA-FLSs via regulating HIPK2. Importantly, we proved that circASH2L could modulate HIPK2 expression via miR-129-5p.

**Conclusion:**

CircASH2L promoted RA-FLS growth, motility, and inflammation through miR-129-5p/HIPK2 axis.

## Introduction

Rheumatoid arthritis (RA) has incredibly high mortality and morbidity rates, impacting around 1% of the population [1, 2]. RA etiology is complex; genetics, epigenetics, and environment are all believed to affect the pathogenesis of RA [3]. Among them, growing findings have indicated the key role of fibroblast-like synoviocytes (FLSs) in the destructive process of RA [4]. FLSs from RA patients (RA-FLSs) display “tumor-like” properties, including excessive proliferation, resistance to apoptosis and increased invasiveness, which result in synovial pannus formation and joint damage [4, 5]. Besides, RA-FLSs stimulate synovial vascularization, which supports the influx of immune cells into affected joints, thus facilitating inflammatory cytokines secretion, ultimately exacerbate the progression of RA [6]. Therefore, a better knowledge on RA-FLS aggressive phenotypes is truly urgent for improving the therapy regimens of RA.

Circular RNAs (circRNAs) are a kind of non-coding transcripts with circular structures, which enable circRNAs resist to RNA degradation pathways and stably represent in eukaryotic cells [7–9]. Existing findings have corroborated that circRNAs involve in managing a variety of cell biological events, including apoptosis, proliferation, invasion, migration, metabolism, inflammation, and vascularization [10, 11], and their dysregulation is correlated with various diseases, like cancers, cardiovascular, and autoimmune diseases [12–14]. Additionally, several circRNAs have been identified as important regulators in RA development. For example, Li et al*.* uncovered that hsa_circ_0001859 expedited inflammatory activity through upregulating transcription factors 2 expression via absorbing miR-204/211 [15]. Circular CDR1 antisense (ciRS-7) relieved miR-7-mediated repression of mTOR, thereby involving in the progression of RA [16]. Recently, circular RNA Absent-Small-Homeotic-2--Like protein (circASH2L, ID: hsa_circ_0083964) was reported to be higher in RA, suggesting the potential influence of circASH2L on the occurrence and development of RA [17].

Herein, this work focused on detecting the expression pattern of circASH2L in RA and RA-FLSs, and explored the role and mechanism of circASH2L with regard to RA-FLS tumor-like biologic behaviors and inflammation, which may help in the understanding of RA pathogenesis.

## Materials and methods

### Clinical specimens and cell culture

The synovial specimens of RA (RA-synovial) were collected from 18 RA patients who received knee synovial debridement or knee joint replacement surgery at Western Theater General Hospital. All patients were diagnosed as RA in line with the American College of Rheumatology classification. The normal synovial specimens (N-synovial) form 18 patients with severe joint trauma were included to serve as controls, and patients with systemic diseases or joint abnormalities were excluded. All specimens were collected from discarded tissues and instantly stored in −80 °C for further analysis. Informed consents have been signed by all subjects, and this study has obtained approval form the Ethics Committee of Western Theater General Hospital.

RA-synovial and N-synovial were cut into small pieces of approximately 1 mm^3^. After washing with Hank’s solution, all pieces were digested by 1 mg/mL collagenase at 37 °C for 3 h to isolate synoviocytes. After that, RA-FLSs and normal FLSs (N-FLSs) were separated using the fluorescence-activated cell sorting (FACS) technique. All collected cells were grown in DMEM containing 10% FBS with 5% CO_2_ atmosphere at 37 °C.

### Cell transfection

The miR-129-5p inhibitor (anti-miR-129-5p) or mimic (miR-129-5p), circASH2L-specific siRNA (si-circASH2L), pcDNA3.1 vector encoding homeodomain-interacting protein kinase 2 (HIPK2), and negative control (anti-miR-NC, miR-NC, si-NC, or vector) were acquired from Invitrogen (Carlsbad, CA, USA). Thereafter, RA-FLSs were transfected with these recombinants (4 μg) and miRNAs mimics or inhibitors (50 nM) using Lipofectamine 2000 (Invitrogen).

### RNase R digestion and qRT-PCR

Total RNAs was obtained by using Trizol reagent. Then treatment with RNase R (3 U/μg) was undertaken at 37 °C for 15 min to detect circRNA. After that, first-strand cDNAs were synthesized with the Reverse Transcription System Kit (Qiagen, Tokyo, Japan), and qPCR was conducted by Power SYBR Green (Qiagen). U6 or GAPDH was used as the internal control, and data was assessed using the 2^−ΔΔCt^ method. Primers for PCR amplification were listed: circASH2L: F 5′-ACCAGTCCATTGGCAAACACT-3′, R 5′-AGGCCCTCACCATAGAGTAGC-3′; HIPK2: F 5′-CCCGTGTACGAAGGTATGGC-3′; R 5′-AGTTGGAACTCGGCTCTATTTTC-3′; ASH2L: F 5′-GGAATTGCAGCAGGAAGCAG-3′, R 5′-GGCGCTGAGCAGAAAACAAA-3′; GADPH: F 5′-GGACCTGACCTGCCGTCTAG-3′, R 5′-TAGCCCAGGATGCCCTTGAG-3′.

### MTT assay

Transfected RA-FLSs (5000 cells/well) were interacted with the MTT reagent (Invitrogen), then DMSO (150 μL) were pipetted into each well to solubilize the MTT. Finally, the absorbance was read to assess cell proliferation at 570 nm.

### 5-Ethynyl-20-Deoxyuridine (EdU) assay

Transfected RA-FLSs were placed into 96-well plates at a density of 5 × 10^4^ cells/well. Then, cell proliferation rated was evaluated using the EdU incorporation assay kit (Keygen, Nanjing, China). A fluorescence microscope was employed to obtain images.

### Flow cytometer

RA-FLSs resuspended in binding buffer were double-stained with 10 μL Annexin V-FITC and PI away from light after assigned transfection, then the apoptotic analysis of RA-FLSs was performed using the flow cytometry.

After transfection, RA-FLSs were in 75% ice-cold ethanol for 24 h, and then stained with FxCycle PI/RNase staining solution (Invitrogen) away from light following the instructions of protocol. At last, cell cycle was quantified using the flow cytometer.

### Western blot

Protein extraction was conducted with RIPA buffer (Beyotime, Shanghai, China) from cells. Then immunoblotting was performed according to standard procedures. The bands of interest were quantified using an ECL Substrate Kit (Tanon, Shanghai, China). All antibodies used were shown as followed: Bcl-2 (1:3000, ab692), Cleaved caspase 3 (c-caspase-3) (1:2000, ab2302), Bax (1:3000, ab32503), HRP-conjugated secondary antibody (1:1000, ab205719), all obtained from Abcam (Cambridge, MA, USA), HIPK2 (1:1000, PA5-14470) obtained from Invitrogen, and β-Actin (1:1,000, 4967) obtained from CST (Boston, MA, USA).

### Transwell assay

Cell invasion or migration capacities were determined using the transwell chambers pre-coated with or without Matrigel (8 μm pore size) (Sangon Biotech, Shanghai, China). Equal numbers of transfected RA-FLSs (1×10^5^ for invasion, 5×10^4^ for migration) with serum-free medium were plated in the upper chambers, lower chamber was filled with medium containing 10% serum. Cells on the lower side were fixed and stained after 24 incubations, and then imaged (×100) and counted using a microscope.

### ELISA assay

After appropriate transfection, the concentrations of TNF-α, IL-1β, and IL-6 from the culture supernatants of RA-FLSs were tested using commercial the ELISA kits.

### Dual-luciferase reporter assay

The specific sequences of circASH2L and HIPK2 3′UTR containing the complementary site of miR-129-5p were annealed into the pmirGLO dual-luciferase vector (Promega, Madison, WI, USA). Then, circASH2L WT/MUT or HIPK2 3′UTR WT/MUT (1 μg) in combination with miR-129-5p or miR-NC (100 nM) were co-transfected into RA-FLSs. At last, the relative luciferase activities were analyzed employing the Dual Luciferase Reporter Assay System.

### Statistical analysis

Data were exhibited as mean ± SD and handled with the GraphPad Prism 7 software. Statistical differences were performed using the Student’s *t* test and one-way ANOVA. *P* < 0.05 suggested statistically significant.

## Results

### CrcASH2L expression profile in RA

**Fig. 1:**
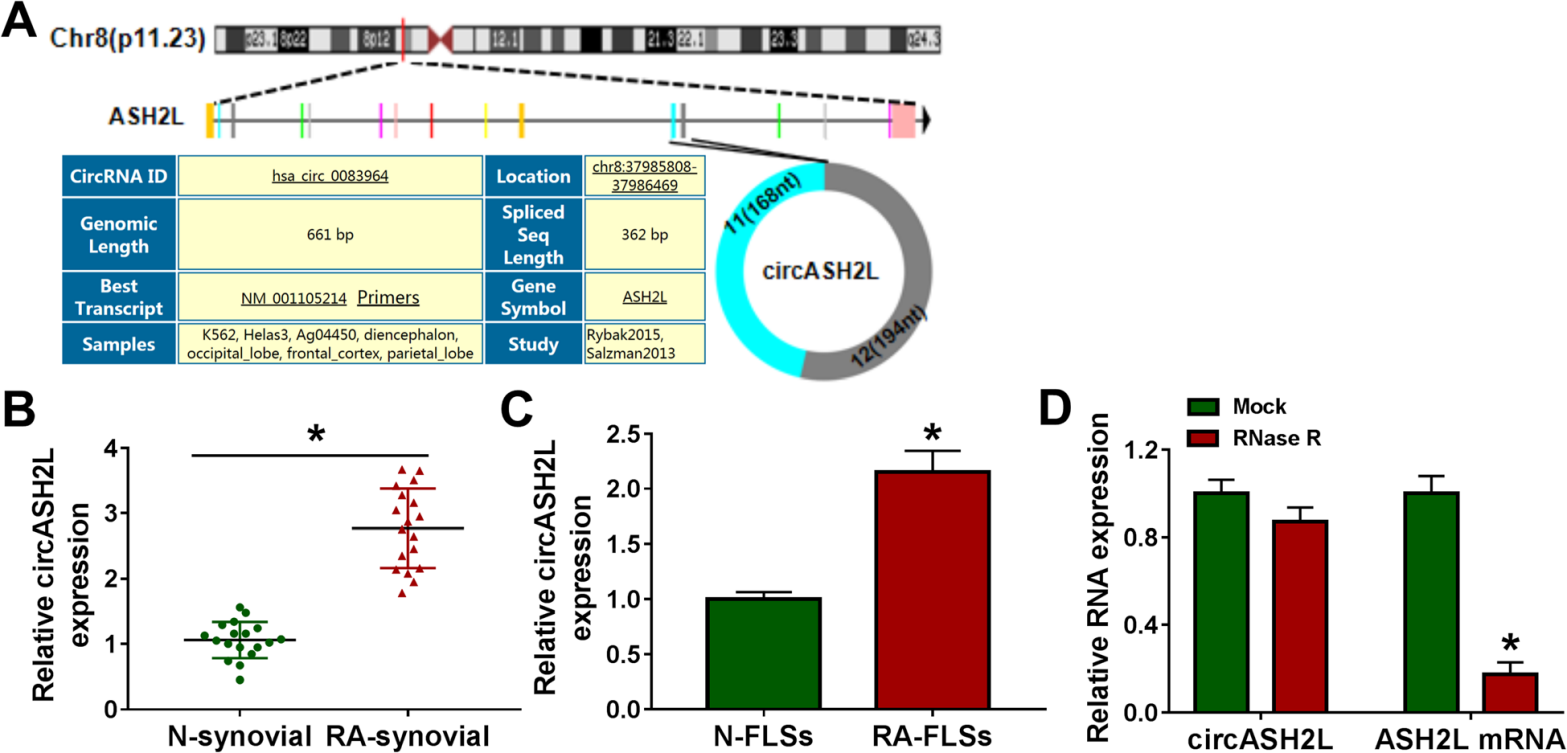
CircASH2L expression in RA. **a** Schematic illustration demonstrates the formation of circASH2L via the circularization of exons 11 and 12 in ASH2L. **b**, **c** Detection of circASH2L expression in synovial tissues with RA and non-RA, as well as in RA-FLSs and N-FLSs with qRT-PCR. **d** Detection of circASH2L and linear ASH2L mRNA expression in RA-FLSs after RNase R treatment using qRT-PCR analysis. *P<0.05

CircASH2L (ID: hsa_circ_0083964) was looped and comprised exons 11 and 12 of its parental gene ASH2L (Fig. 1a). Then, the expression profile of circASH2L in RA was investigated adopting qRT-PCR assay. It was observed that circASH2L was expressively elevated in synovial tissues with RA in comparison to these in normal synovial specimens (N-synovial) (Fig. 1b). Also, its expression was elevated in RA-FLSs compared with normal FLSs (N-FLSs) (Fig. 1c). Additionally, qRT-PCR analysis also suggested circASH2L, but not linear ASH2L mRNA, could resist RNase R digestion in RA-FLSs (Fig. 1d), confirming that circASH2L was indeed circular. Therefore, all these data suggested that the aberrant circASH2L expression might be associated with the progression of RA.

### Effects of circASH2L on the tumor-like biologic behaviors of RA-FLSs

**Fig. 2:**
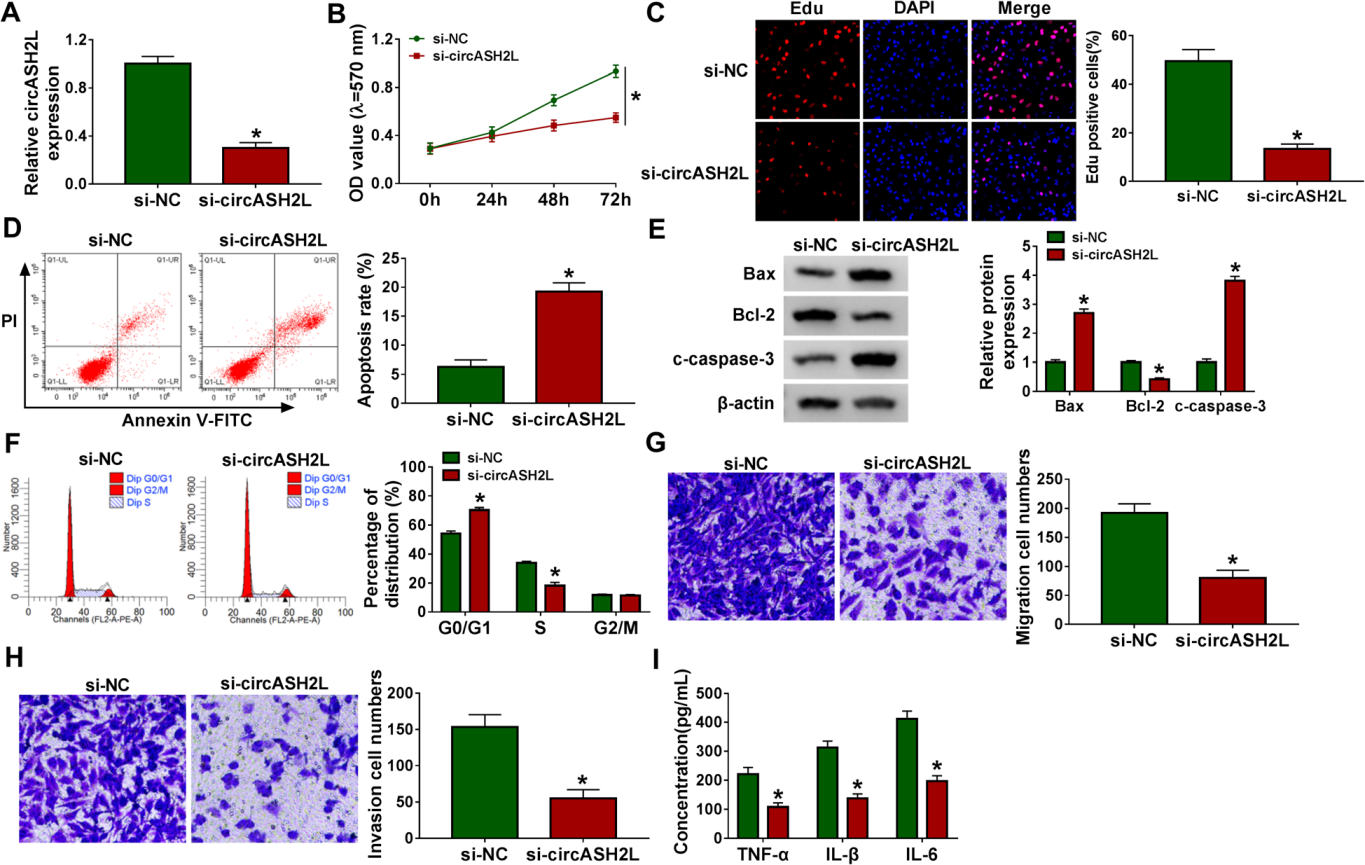
Effects of circASH2L on the tumor-like biologic behaviors of RA-FLSs. **a**-i si-circASH2L or si-NC was transfected into RA-FLSs, respectively. **a** Measurement of circASH2L expression in RA-FLSs with qRT-PCR. **b**, **c** Cell proliferation analysis with MTT assay and Edu assay. **d** Flow cytometry for cell apoptosis. **e** Detection of apoptosis-related protein using western blot. **f** Flow cytometry of cell cycle analysis. **g**, **h** Cell migration and invasion analysis with transwell assay. **i** Detection of inflammatory cytokines expression using ELISA. *P<0.05

Next, the action of circASH2L in RA progression was studied. The si-circASH2L was used to knockdown circASH2L in RA-FLSs, as expected, circASH2L expression was significantly reduced in si-circASH2L-transfected RA-FLSs (Fig. 2a). After that, we found that circASH2L silencing suppressed cell proliferation in RA-FLSs, as MTT assay and Edu assay indicated (Fig. 2b, c). Conversely, the apoptosis of RA-FLSs was remarkably promoted by the downregulation of circASH2L, evidenced by the increase of apoptotic cells (Fig. 2d), Bax, c-caspase-3 protein expression, and decreased of Bcl-2 expression (Fig. 2e) in the si-circASH2L group. Meanwhile, results from flow cytometry also revealed that circASH2L silencing induced the increase of the proportions of cells in G0/G1 phase and decrease in S phase, inducing cell cycle arrest (Fig. 2f). Thus, knockdown of circASH2L had an inhibitory effect on RA-FLS growth. Additionally, transwell assay results showed a prominent reduction of cell migratory and invasive capacities in circASH2L-decraesed RA-FLSs (Fig. 2g, h). Furthermore, it was also discovered that the secretion of IL-6, TNF-α, and IL-1β was suppressed by the introduction of si-circASH2L in RA-FLSs (Fig. 2i). Taken together, circASH2L knockdown repressed the growth, motility, and inflammation of RA-FLSs.

### MiR-129-5p is a target of circASH2L in RA-FLSs

**Fig. 3:**
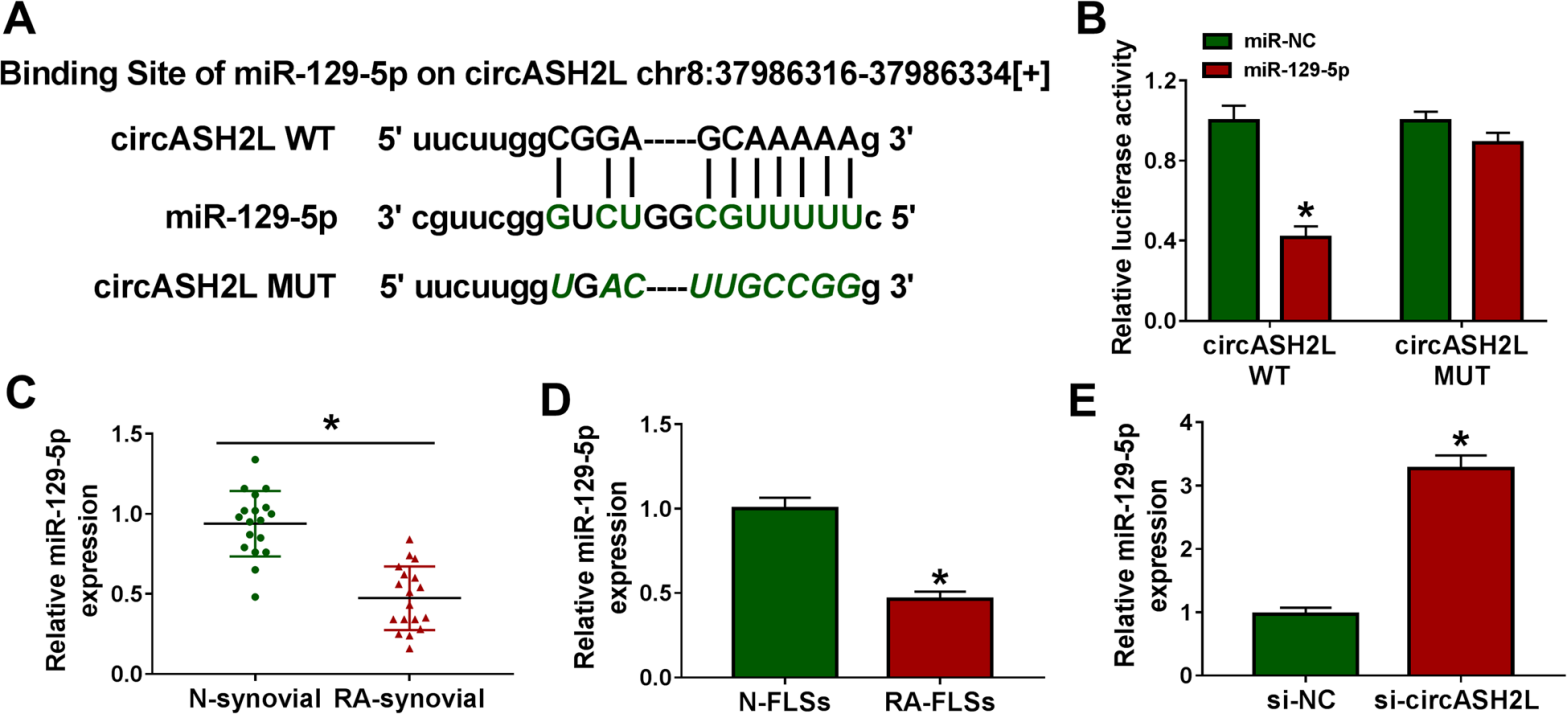
CircASH2L directly bind to miR-129-5p in RA-FLSs. **a** Binding sites between miR-129-5p and circASH2L. **b** Interaction analysis between miR-129-5p and circASH2L using the dual-luciferase reporter assay. **c**, **d** MiR-129-5p expression in synovial tissues with RA and non-RA, as well as in RA-FLSs and N-FLSs was examined by qRT-PCR. **e** qRT-PCR analysis of miR-129-5p expression in circASH2L-decreased RA-FLSs. *P<0.05

To elucidate circRNA-microRNA (miRNA) interaction potentials, the bioinformatics method (starBase v2.0) were employed and miR-129-5p was found to have complementary binding sites in circASH2L (Fig. 3a). Afterwards, the dramatically reduction of the luciferase activity in RA-FLSs co-transfected with circASH2L WT or miR-129-5p mimic confirmed the direct interaction between circASH2L and miR-129-5p (Fig. 3b). Thereafter, a decreased miR-129-5p expression was detected in RA synovial tissues and RA-FLSs by contrast with normal controls (Fig. 3c, d). Importantly, it was proved that circASH2L silencing increased miR-129-5p level in RA-FLSs (Fig. 3e). Collectively, we confirmed that circASH2L directly targeted miR-129-5p and suppressed its level in RA-FLSs.

### Knockdown of circASH2L suppresses the tumor-like biologic behaviors of RA-FLSs through miR-129-5p

**Fig. 4:**
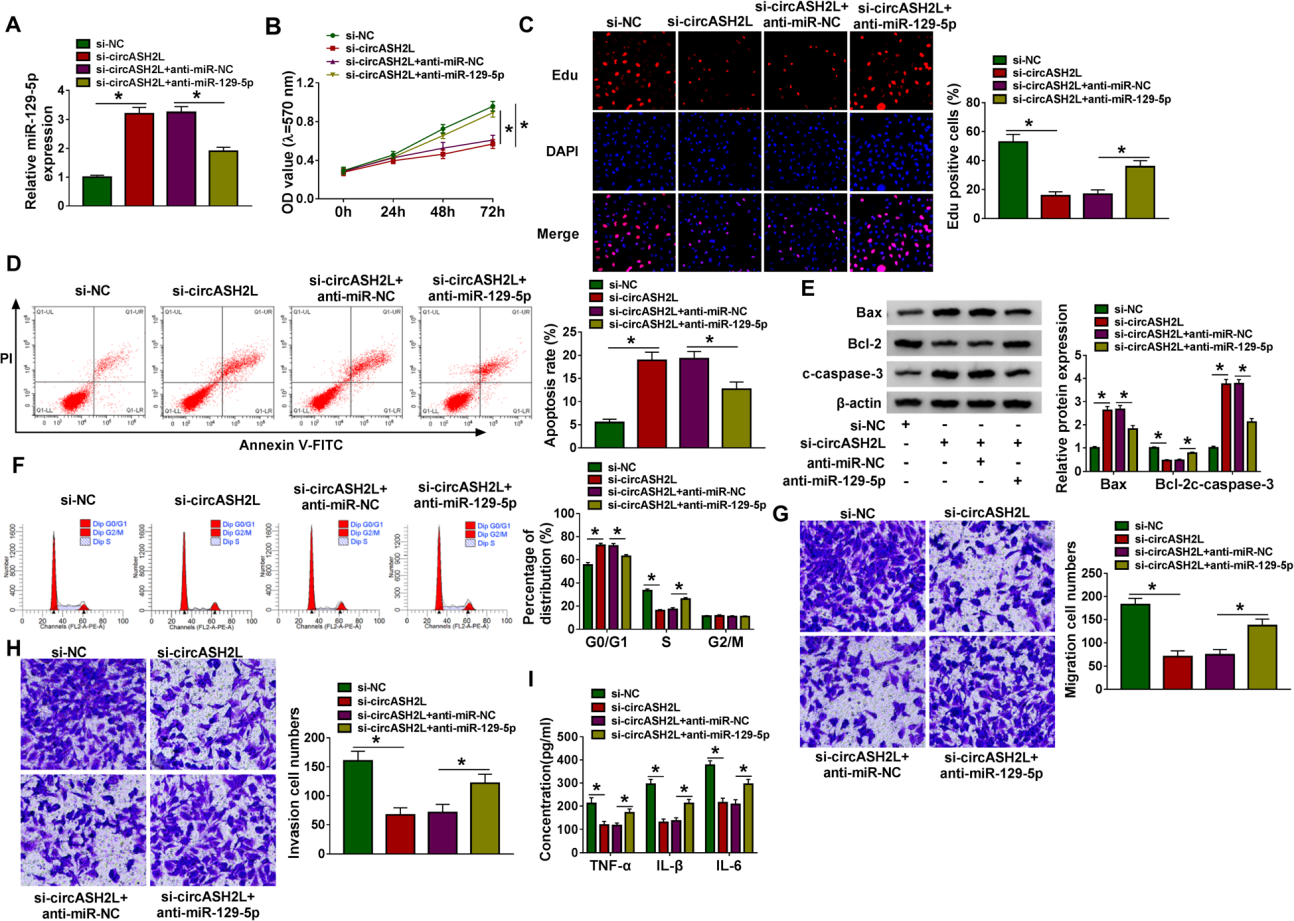
Knockdown of circASH2L suppresses the tumor-like biologic behaviors of RA-FLSs through miR-129-5p. **a**-**i** RA-FLSs were co-transfected with si-circASH2L and/or anti-miR-129-5p. **a** qRT-PCR analysis of miR-129-5p expression in RA-FLSs. **b**, **c** Cell proliferation analysis using MTT assay and Edu assay. **d** Flow cytometry of cell apoptosis analysis. **e** Detection of apoptosis-related protein expression using western blot. **f** Flow cytometry of cell cycle analysis. **g**, **h** The migratory and invasive activities analysis of cells using transwell assay. **i**) The measurement of inflammatory cytokines levels using ELISA. *P<0.05

To elucidate whether si-circASH2L-induced inhibitory effects was dependent on miR-129-5p, miR-129-5p inhibitor was introduced into si-circASH2L-transfected RA-FLSs, and miR-129-5p repression significantly reduced si-circASH2L-induced elevation of miR-129-5p level in RA-FLSs as expected (Fig. 4a). Then, we discovered the introduction of miR-129-5p inhibitor notably impaired si-circASH2L-mediated repression of cell proliferation (Fig. 4b, c), promotion of cell apoptosis (Fig. 4d, e), arrest of cell cycle (Fig. 4f), decrease of cell migration and invasiveness (Fig. 4g, h), and inhibition of inflammation (Fig. 4i) in RA-FLSs. Altogether, circASH2L knockdown reduced RA development via miR-129-5p.

### CircASH2L indirectly regulates HIPK2, the target of miR-129-5p, via targeting miR-129-5p in RA-FLSs

**Fig. 5:**
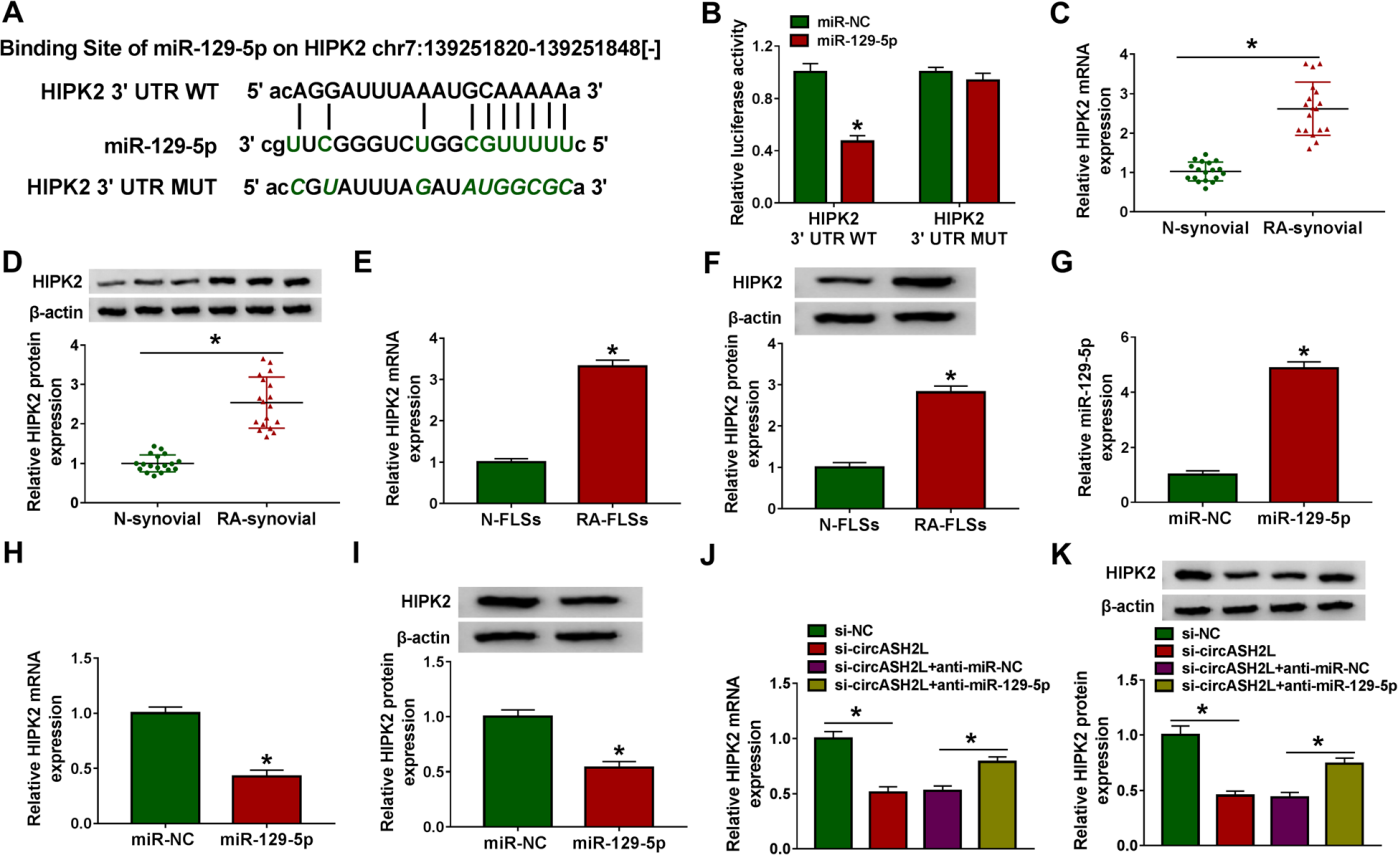
CircASH2L indirectly regulates HIPK2 via miR-129-5p in RA-FLSs. **a** Binding sites of miR-129-5p on HIPK2 3′UTR. **b** Interaction analysis between miR-129-5p and HIPK2 using the dual-luciferase reporter assay. **c**-**f** HIPK2 expression level in synovial tissues with RA and non-RA, as well as in RA-FLSs and N-FLSs was determined. **g** MiR-129-5p expression analysis using qRT-PCR in RA-FLSs transfected with miR-NC or miR-129-5p. **h**, **i** HIPK2 expression analysis in miR-129-5p-increased RA-FLSs with qRT-PCR and western blot. **j**, **k** RA-FLSs were co-transfected with si-circASH2L and/or anti-miR-129-5p and HIPK2 level was examined using qRT-PCR and western blot. *P<0.05

We then further studied the possible molecular mechanisms that were responsible for the action of miR-129-5p in RA development. Through starBase v2.0 online database, miR-129-5p also was found to have complementary binding sites in HIPK2 (Fig. 5a). Then, it was proved that miR-129-5p overexpression declined the luciferase activity of HIPK2 3′UTR WT reporter, but not the mutant one in RA-FLSs, revealing that miR-129-5p targeted HIPK2 (Fig. 5b). HIPK2 expression was found to be higher in RA synovial tissues and RA-FLSs (Fig. 5c-f); meanwhile, when we elevated miR-129-5p level by transfecting miR-129-5p mimic into RA-FLSs (Fig. 5g), the expression of HIPK2 showed a marked decrease in miR-129-5p-upregulated RA-FLSs (Fig. 5h, i). All these data validated that miR-129-5p targetedly modulated HIPK2 expression in RA-FLSs.

Besides that, we also found knockdown of circASH2L weaken the expression of HIPK2 in RA-FLSs, which was rescued by miR-129-5p inhibition (Fig. 5j, k), indicating circASH2L could indirectly regulate HIPK2 expression via miR-129-5p.

### MiR-129-5p impairs RA-FLS tumor-like biologic behaviors through HIPK2

**Fig. 6:**
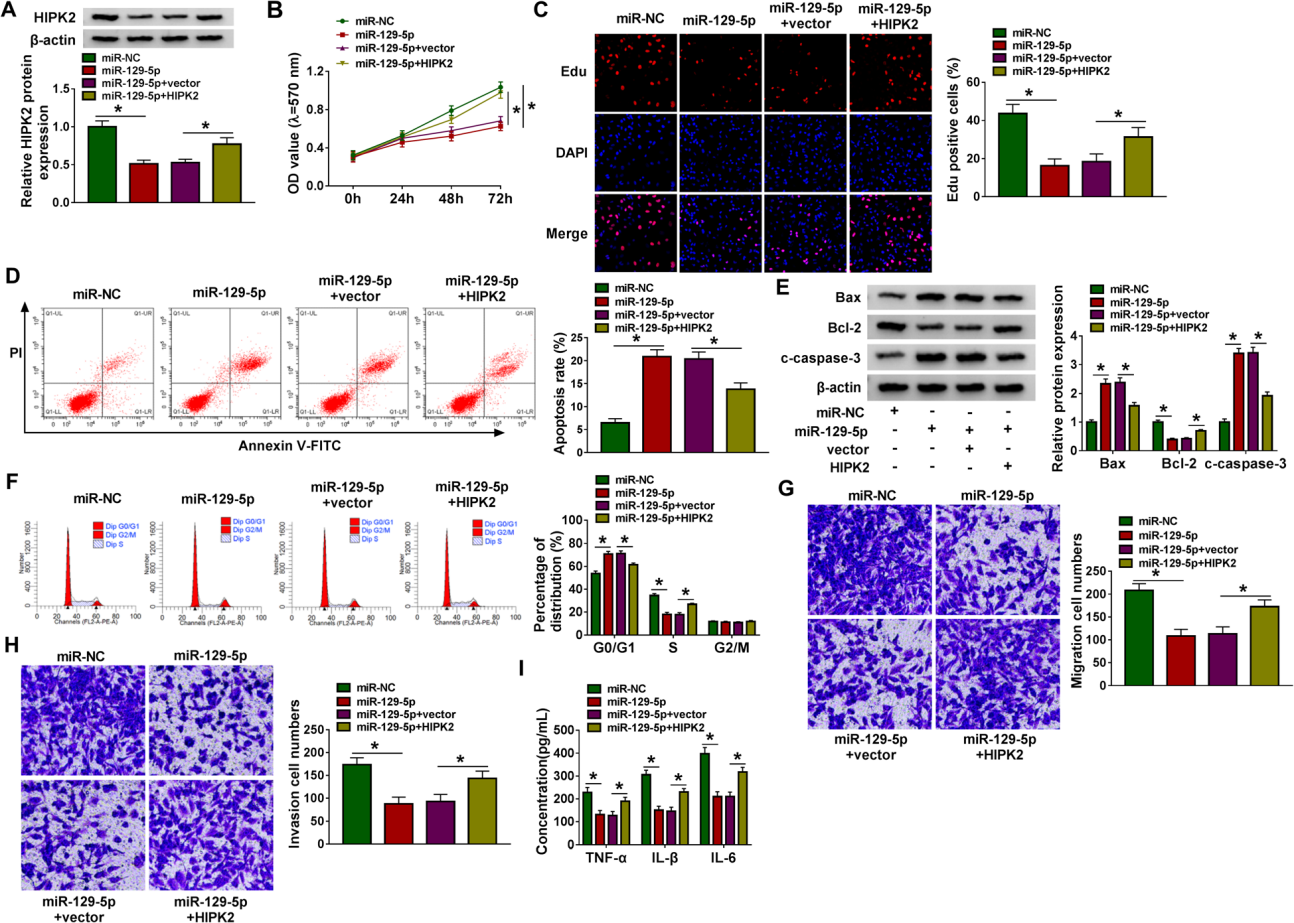
MiR-129-5p impairs RA-FLS tumor-like biologic behaviors through HIPK2. The miR-129-5p and/or HIPK2 were co-transfected into RA-FLSs. After transfection, **a** western blot analysis of HIPK2 expression using western blot; **b**, **c** MTT assay of cell proliferation and Edu assay; **d** flow cytometry of cell apoptosis detection; **e** western blot analysis of apoptosis-related protein expression in cells; **f** flow cytometry for cell cycle analysis; **g**, **h** cell migratory and invasive abilities detection using transwell assay; **i** the measurement of inflammatory cytokines levels using ELISA. *P<0.05

Next, we attempted to probe whether FGFR1 was the functional target of miR-129-5p in inhibiting RA progression. The miR-129-5p and/or HIPK2 were co-transfected into RA-FLSs, then it was observed that HIPK2 rescued miR-129-5p-stimulated decrease of HIPK2 expression in cells (Fig. 6a), suggesting the successful interference. After that, MTT assay, flow cytometry, and western blot were carried out. Results indicated miR-129-5p re-expression in RA-FLSs suppressed cell proliferation (Fig. 6b, c), promoted apoptosis (Fig. 6d, e), and evoked cell cycle arrest (Fig. 6f); however, these effects were partially overturned by HIPK2 overexpression (Fig. 6b-f). In addition, transwell assay showed that the anti-motility roles of miR-129-5p restoration on RA-FLSs could be abolished by HIPK2 upregulation (Fig. 6g, h). Moreover, results of ELISA exhibited that HIPK2 overexpression abated the repressive functions of miR-129-5p mimic on RA-FLSs inflammation, reflected by the increase of TNF-α, IL-6, and IL-1β levels in cells (Fig. 6i). Thus, we demonstrated that miR-129-5p hindered RA progression through regulating HIPK2.

## Discussion

Currently, increasing evidence has indicated that FLSs in the synovial intimal lining contribute to cartilage destruction by producing cytokines to perpetuate inflammation; besides that, RA-FLSs actively increase invasiveness into articular cartilage, positively inducing the expression of adhesion molecules and proinflammatory and matrix-degrading mediators, which in turn exacerbate joint damage [6, 18]. Therefore, studies on the mechanisms that control RA-FLSs behavior may help further to elucidate the pathogenesis of RA.

CircRNAs are a kind of RNAs that show high stability, evolutionary conservation, and tissue-specific expression, which render them suitable for further development into putative biomarkers of various diseases [19, 20], besides, they play key regulatory roles in multiple cellular processes [10]. Lately, deregulation of circRNAs in RA was revealed to be functionally associated with RA progression [21–23]. In this study, circASH2L expression showed a significant increase in RA, after knocking down the expression of circASH2L in RA-FLSs using siRNA, and found circASH2L downregulation repressed cell proliferation, invasion, migration, cell cycle progression, triggered cell apoptosis, and prevented inflammation in RA-FLSs. Therefore, knockdown of circASH2L might impair the progression of RA through suppressing RA-FLS growth, motility, and inflammation.

Previous findings have documented that circRNAs can function as sponges of miRNAs to control gene expression [20, 24]. The circRNA-miRNA-mRNA axis has been revealed to implicate in a variety of signaling cascades, such as those associated with the growth, apoptosis, vascularization, and invasion [25]. Thus, the regulatory network underlying circASH2L in RA-FLSs was investigated. This study confirmed that circASH2L directly targeted miR-129-5p. One previous study displayed that miR-129-5p was decreased in RA, which upregulation in RA-FLSs induced cell proliferation inhibition and apoptosis promotion [26]. In this work, a decreased miR-129-5p in RA was also observed, besides that, its re-expression also suppressed cell growth. In the meanwhile, we also proved that miR-129-5p restoration destroyed cell growth, motility, and inflammation in RA-FLSs. Importantly, miR-129-5p repression partially overturned the inhibitory effects of si-circASH2L on RA-FLS tumor-like biologic behaviors and inflammation.

In the current work, we also verified that miR-129-5p directly targeted HIPK2 in RA-FLSs; moreover, circASH2L could indirectly regulate HIPK2 by targeting miR-129-5p. HIPK2 belongs to the HIPKs family, and can be categorized as a serine/threonine protein kinase, which involves in transcription modulation, p53/TP53-mediated cellular apoptosis, and cell cycle regulation [27, 28]. Previous researches have showed HIPK2 was enriched in RA patients and RA-FLSs [29, 30], and participated in the GAS5-mediated repression of the inflammatory response and proliferation in RA-FLSs [30]. This study also showed a high expression of HIPK2 in RA; more importantly, overexpression of HIPK2 abrogated the action of miR-129-5p in RA-FLS tumor-like biologic behaviors.

In summary, this study demonstrated that circASH2L promoted RA-FLS tumor-like behaviors and inflammation via miR-129-5p/HIPK2 axis, extending current knowledge on RA pathogenesis. Currently, there are more than 20 clinical trials that are underway evaluating the potential utility of RNA interference (RNAi)-based therapeutics in various human diseases, six RNAi-based therapeutic agents using synthetic siRNAs or bifunctional shRNA have progressed into phase 3 clinical trials [31]. Therefore, the synthetic siRNAs targeting circASH2L may be candidates for the development of therapeutic methods for RA patients. However, the expression and function of siRNAs in healthy cell lines should be analyzed before the application in clinical to ensure the safety and efficiency. Besides that, the delivery method, limited oral bioavailability, and rapid clearance rate from circulation of siRNAs remain the major challenges for the development of RNAi-based therapeutics.

## Data Availability

Not applicable

## Abbreviations

circASH2L: Circular RNA Absent-Small-Homeotic-2--Like protein
RA: Rheumatoid arthritis
FLSs: Fibroblast-like synoviocytes
FACS: Fluorescence activated cell sorting

## References

[1] McInnes IB, Schett G (2017). Pathogenetic insights from the treatment of rheumatoid arthritis. Lancet (London, England).

[2] Covic T, Cumming SR, Pallant JF, Manolios N, Emery P, Conaghan PG, Tennant A (2012). Depression and anxiety in patients with rheumatoid arthritis: prevalence rates based on a comparison of the Depression, Anxiety and Stress Scale (DASS) and the hospital, Anxiety and Depression Scale (HADS). BMC psychiatry.

[3] Croia C, Bursi R, Sutera D, Petrelli F, Alunno A, Puxeddu I (2019). One year in review 2019: pathogenesis of rheumatoid arthritis. Clinical and experimental rheumatology.

[4] Neumann E, Lefèvre S, Zimmermann B, Gay S, Müller-Ladner U (2010). Rheumatoid arthritis progression mediated by activated synovial fibroblasts. Trends in molecular medicine.

[5] Bottini N, Firestein GS (2013). Duality of fibroblast-like synoviocytes in RA: passive responders and imprinted aggressors. Nature reviews Rheumatology.

[6] Lefevre S, Meier FM, Neumann E, Muller-Ladner U (2015). Role of synovial fibroblasts in rheumatoid arthritis. Current pharmaceutical design.

[7] Rybak-Wolf A, Stottmeister C, Glažar P, Jens M, Pino N, Giusti S, Hanan M, Behm M, Bartok O, Ashwal-Fluss R, Herzog M, Schreyer L, Papavasileiou P, Ivanov A, Öhman M, Refojo D, Kadener S, Rajewsky N (2015). Circular RNAs in the mammalian brain are highly abundant, conserved, and dynamically expressed. Molecular cell.

[8] Memczak S, Jens M, Elefsinioti A, Torti F, Krueger J, Rybak A, Maier L, Mackowiak SD, Gregersen LH, Munschauer M (2013). Circular RNAs are a large class of animal RNAs with regulatory potency. Nature.

[9] Zhou R, Wu Y, Wang W, Su W, Liu Y, Wang Y, Fan C, Li X, Li G, Li Y, Xiong W, Zeng Z (2018). Circular RNAs (circRNAs) in cancer. Cancer letters.

[10] Yu CY, Kuo HC (2019). The emerging roles and functions of circular RNAs and their generation. Journal of biomedical science.

[11] Marques-Rocha JL, Samblas M, Milagro FI, Bressan J, Martínez JA, Marti A (2015). Noncoding RNAs, cytokines, and inflammation-related diseases. FASEB journal : official publication of the Federation of American Societies for Experimental Biology.

[12] Vo JN, Cieslik M, Zhang Y, Shukla S, Xiao L, Zhang Y, Wu YM, Dhanasekaran SM, Engelke CG, Cao X (2019). The landscape of circular RNA in cancer. Cell.

[13] Altesha MA, Ni T, Khan A, Liu K, Zheng X (2019). Circular RNA in cardiovascular disease. Journal of cellular physiology.

[14] Chen X, Yang T, Wang W, Xi W, Zhang T, Li Q, Yang A, Wang T (2019). Circular RNAs in immune responses and immune diseases. Theranostics.

[15] Li B, Li N, Zhang L, Li K, Xie Y, Xue M, Zheng Z (2018). Hsa_circ_0001859 regulates ATF2 expression by functioning as an MiR-204/211 sponge in human rheumatoid arthritis. J Immunology Res.

[16] Tang X, Wang J, Xia X, Tian J, Rui K, Xu H, Wang S (2019). Elevated expression of ciRS-7 in peripheral blood mononuclear cells from rheumatoid arthritis patients. Diagnostic pathology.

[17] Zheng F, Yu X, Huang J, Dai Y (2017). Circular RNA expression profiles of peripheral blood mononuclear cells in rheumatoid arthritis patients, based on microarray chip technology. Molecular medicine reports.

[18] Bartok B, Firestein GS (2010). Fibroblast-like synoviocytes: key effector cells in rheumatoid arthritis. Immunological reviews.

[19] Jeck WR, Sharpless NE (2014). Detecting and characterizing circular RNAs. Nature biotechnology.

[20] Jin Y, Shenoy AK, Doernberg S, Chen H, Luo H, Shen H, Lin T, Tarrash M, Cai Q, Hu X, Fiske R, Chen T, Wu L, Mohammed KA, Rottiers V, Lee SS, Lu J (2015). FBXO11 promotes ubiquitination of the Snail family of transcription factors in cancer progression and epidermal development. Cancer letters.

[21] Luo Q, Liu J, Fu B, Zhang L, Guo Y, Huang Z, Li J (2019). Circular RNAs Hsa_circ_0002715 and Hsa_circ_0035197 in peripheral blood are novel potential biomarkers for new-onset rheumatoid arthritis. Disease markers.

[22] Luo Q, Zhang L, Li X, Fu B, Deng Z, Qing C, Su R, Xu J, Guo Y, Huang Z, Li J (2018). Identification of circular RNAs hsa_circ_0044235 in peripheral blood as novel biomarkers for rheumatoid arthritis. Clinical and experimental immunology.

[23] Ouyang Q, Wu J, Jiang Z, Zhao J, Wang R, Lou A, Zhu D, Shi GP, Yang M (2017). Microarray expression profile of circular RNAs in peripheral blood mononuclear cells from rheumatoid arthritis patients. Cellular physiology and biochemistry : international journal of experimental cellular physiology, biochemistry, and pharmacology.

[24] Hansen TB, Jensen TI, Clausen BH, Bramsen JB, Finsen B, Damgaard CK, Kjems J (2013). Natural RNA circles function as efficient microRNA sponges. Nature.

[25] Chien Y, Tsai PH, Lai YH, Lu KH, Liu CY, Lin HF, Huang CS, Wu WW, Wang CY (2020). CircularRNA as novel biomarkers in liver diseases. Journal of the Chinese Medical Association : JCMA.

[26] Zhang Y, Yan N, Wang X, Chang Y, Wang Y (2019). MiR-129-5p regulates cell proliferation and apoptosis via IGF-1R/Src/ERK/Egr-1 pathway in RA-fibroblast-like synoviocytes. Bioscience Rep.

[27] Saul VV, Schmitz ML (2013). Posttranslational modifications regulate HIPK2, a driver of proliferative diseases. J Mol Med (Berlin, Germany).

[28] Blaquiere JA, Verheyen EM (2017). Homeodomain-interacting protein kinases: diverse and complex roles in development and disease. Curr Topics Dev Biol.

[29] Li Y, Lai-Han Leung E, Pan H, Yao X, Huang Q, Wu M, Xu T, Wang Y, Cai J, Li R, Liu W, Liu L (2017). Identification of potential genetic causal variants for rheumatoid arthritis by whole-exome sequencing. Oncotarget.

[30] Li M, Wang N, Shen Z, Yan J. Long non-coding RNA growth arrest-specific transcript 5 regulates rheumatoid arthritis by targeting homeodomain-interacting protein kinase 2. Clin Experimental Rheumatology. 2020. 32141429

[31] Sullenger BA, Nair S (2016). From the RNA world to the clinic. Science (New York, NY).

